# Prenatal symptoms of anxiety and depression associated with sex differences in both maternal perceptions of one year old infant temperament and researcher observed infant characteristics

**DOI:** 10.1016/j.jad.2019.11.057

**Published:** 2020-03-01

**Authors:** K. Savory, S.M. Garay, L.A. Sumption, J.S. Kelleher, K. Daughters, A.B. Janssen, S. Van Goozen, R.M. John

**Affiliations:** a Biomedicine Division, School of Biosciences, Cardiff University, Cardiff, CF10 3AX United Kingdom; b Neuroscience and Mental Health Research Institute, Cardiff University, Cardiff, CF24 4HQ United Kingdom; c Cardiff University Centre for Human Developmental Science, School of Psychology, Cardiff University, Cardiff, CF10 3AT United Kingdom

**Keywords:** Maternal mood symptoms, Perception bias, Sex differences, Bonding, Aggression, Negative affectivity

## Abstract

**Background:**

Sex differences in the behaviour of children exposed to prenatal maternal depression and anxiety have been reported. This study compared depression and anxiety symptoms reported by mothers at term with maternal perceptions of one year old male and female infant temperament and with researcher observed infant characteristics, identifying differences for males and females with both approaches.

**Methods:**

Infant behaviour and temperament was assessed via maternally completed questionnaires including Infant Behavioural Questionnaire Revised – Short form and by researcher administered subcomponents of Laboratory Temperament Assessment Battery and Bayley Scales of Infant Development III.

**Results:**

For female infants, higher prenatal scores for depression and anxiety were associated with maternal perceptions of lower bonding, higher aggression and negativity, and lower soothability (*n* = 67 mother-infant dyads). In the laboratory assessment, intensity of escape was the only female infant factor significantly associated with maternal mood (*n* = 41). For male infants, there was minimal association between prenatal mood scores and maternal perceptions (*n* = 46) whereas in the laboratory assessment (*n* = 35) depression scores were associated with expressive language, facial interest and facial fear while anxiety scores were associated with expressive and receptive language, parent behaviour and facial fear.

**Limitations:**

Findings may be restricted to a single ethnicity or mode of delivery. Fewer infants attended the infant assessment. A laboratory setting may mask symptomatology in females.

**Conclusions:**

Atypical maternal perceptions may present a barrier to the early identification of male infants impacted by maternal depression and anxiety.

## Highlights

• Maternal perceptions of temperament influenced by mood and infant gender.
• Mood affected mothers report poor bonding and difficulties in daughters, not sons.
• Researchers observe minimal difficulties in girls exposed to maternal symptoms.
• Researchers observe language delay and temperament issues in exposed boys.
• Maternal perceptional biases may blind mothers to problems in their sons.

## 1 Introduction

One in seven pregnancies are impacted by maternal depression and over one quarter are impacted by concerning levels of anxiety (Heron et al., 2004; Janssen et al., 2018; Lockwood Estrin et al., 2019). Depression and anxiety in pregnancy are associated with low birth weight (Berkowitz et al., 2003; Dunkel Schetter and Tanner, 2012; Henrichs et al., 2010; Khashan et al., 2008; Liu et al., 2012; Paarlberg et al., 1999; Pritchard and Teo, 1994; Steer et al., 1992; Uguz et al., 2013), and difficulties in infant development including emotional and behavioural problems, cognitive impairment and psychopathology (Lahti et al., 2017; Raikkonen et al., 2015; Talge et al., 2007; Van den Bergh et al., 2005). Despite considerable epidemiological data reporting these links between exposure and outcomes, the underpinning biological mechanisms are unknown nor can we predict specific outcomes. Progress is hampered because the causes and consequences of maternal mood disorders are complex, with multiple environmental and genetic components, exposure is both prenatal and postnatal and there is a reliance on self-reported information not always validated by independent, objective observations. A further confounder is data suggesting sex differences in the response to stressors during development and after birth (Hicks et al., 2019). Studies exploring the relationship between maternal mood symptoms and outcomes generally control for sex in the analysis and aim to have an equal representation of males and females. This approach may obscure sex-specific findings adding further complexity.

### 1.1 Sex differences in maternally-reported infant temperament

Although few studies have analysed males and females as distinct entities, those that have made this distinction report differences in the cognitive and behavioural outcomes of male and female children exposed to maternal mood symptoms. Exposure to maternal depression has been associated with a higher prevalence of maternally reported emotional disorders including dysphoric mood, anxiety and obsessive-compulsiveness in daughters but not sons (Boyle and Pickles, 1997); poorer cognitive function and lower educational attainment specifically for teenage boys (Murray et al., 2010); insecure attachment for boys and girls at greater risk of anxiety as teenagers (Murray et al., 2011); and higher internalising scores for boys and girls aged 24 months with higher externalising scores only for girls (Soe et al., 2016). The strong relationship between depression in pregnancy and postnatal depression symptoms (Heron et al., 2004) means that many studies cannot attribute their findings to the prenatal or postnatal exposure, or their combined impact. However, Quarini et al., reported a correlation between prenatal depression and higher levels of depression in older teenage girls and between postnatal depression and higher levels of depression in older teenage boys (Quarini et al., 2016) suggesting that the timing of exposure can contribute to sexually dimorphic outcomes.

Depression is commonly comorbid with anxiety (Glover, 2014) and children exposed to maternal anxiety symptoms show similar sexually dimorphic outcomes such as attention deficits, cognitive problems and externalising behaviour in boys and with anxiety, depression and internalising symptoms in girls (Glover and Hill, 2012). For example, maternal anxiety has been associated with child behavioural problems at four years with both sexes exhibiting behavioural and emotional problems, and boys specifically displaying higher levels of hyperactivity and inattention based on maternally-reported questionnaires (O'Connor et al., 2002); depression in adolescent girls but not boys (Van den Bergh et al., 2008); and differences in the attention patterns and vigilance depending on the sex of the infant (Kataja et al., 2019). Prenatal stress, defined as perceptions of situations as threatening, unpredictable or uncontrollable, has been linked to attention deficit and hyperactivity disorder (ADHD) symptoms more strongly in boys (Rodriguez and Bohlin, 2005). Most recently, maternal depression and anxiety symptoms only in the prenatal period have been associated with higher observed fear in 8 month female but not male infants (Nolvi et al., 2019).

### 1.2 Sex differences in biological measures

Independent, biological measures that provide objective data have provided additional evidence for sex-specific characteristics. The stress hormone cortisol can be used as a proxy for maternal psychosocial stress (Hellhammer et al., 2009). High maternal salivary levels of cortisol late in pregnancy have been linked to maternal reports of increased irritability in five week old female infants, and decreased irritability in males (Braithwaite et al., 2017b). Although the link between maternal cortisol and maternal anxiety in pregnant women is not straightforward (Garay et al., 2019; Harville et al., 2009; Janssen et al., 2018; van den Heuvel et al., 2018), these same researchers reported a similar sex-specific relationship between a second biological marker, maternal salivary alpha-amylase, and sex-specific indications of negative emotionality in infants (Braithwaite et al., 2017a). Brain structure ascertained through magnetic resonance imaging provides another biological measure that does not rely on parental reporting. Sex-specific structural differences have been reported in children exposed in utero to depression and/or anxiety. Depression in pregnancy has been linked to a higher right amygdala volume in 4.5 year old daughters but not sons (Wen et al., 2017). Higher maternal cortisol levels in early pregnancy have been linked to an increased amygdala volume and maternal reporting of affective problems in seven year old daughters and not sons (Buss et al., 2012). Depression in the second trimester of pregnancy has been linked to reduced cortical thickness in girls and depression after birth has been linked to lower diffusivity in the white matter of boys assessed two to five years after birth (Lebel et al., 2016). This finding has essentially been reproduced in a second study (Dean et al., 2018). These findings support sexually dimorphic outcomes for male and female children exposed to maternal mental health problems early in life.

## 2 The current study

While sex-specific differences in infant temperament have been reported by mothers with mental health problems in pregnancy, depression in particular is thought to alter aspects of perception (Kornbrot et al., 2013). Depressed or anxious mothers may perceive difficulties with their infants due to their mood symptoms (Webb and Ayers, 2015). To our knowledge, no study has explored the possibility that maternal mood symptoms are associated with sex-specific differences in maternal perceptions of her child's temperamental characteristics that do not concur with independently observed child behaviour. In this study, we aimed to address this gap in our knowledge by undertaking a maternally-completed questionnaire approach alongside objectively measured laboratory-based observations of development and temperament of the same children to obtain independent evidence for sex-differences. We analyse data for one year old infants in relation to prenatal mental health symptoms self-reported at term by women participating in The Grown in Wales (GiW) Study (Janssen et al., 2018).

## 3 Methods

### 3.1 Participants

Full ethical approval for the ongoing pregnancy cohort study was obtained via the Wales Research Ethics Committee reference 15/WA/0004. The GiW cohort has been previously described (Janssen et al., 2018). Briefly, the GiW cohort is a longitudinal study in the South Wales region of the United Kingdom. Women were recruited to the study to examine the relationship between prenatal mood symptoms, placental genomic characteristics and offspring outcomes. Recruitment was undertaken by two trained research midwives at the University Hospital of Wales (UHW) between 1st September 2015 and 31st November 2016 at a presurgical appointment prior to a booked elective caesarean section (ELCS) if they met the criteria of a singleton term pregnancy without fetal anomalies and infectious diseases. Women provided written consent and 355 were recruited to the cohort, and seven later withdrew. Demographic data for the overall cohort recruited in pregnancy excluding those withdrawn (n = 348) are provided in Table 1 for reference. ELCS delivery was chosen as the single mode of delivery for the purposes of collecting biological samples, work that will be described elsewhere.

**Table 1:** Demographic data for the Grown in Wales Study participants and those completing questionaires and assessment.

Demographics	% (n) or median (IQR)		
	Grown in Wales Study (348)	Questionnaire Reponders (113)	Assessment Attendees (76)
Maternal age at Year 1	N/A	36.00 (7.00)	36.00 (6.00)
Parity, *% (n)*^a^			
*Nulliparous*	19.60 (68)	26.50 (30)	25.00 (19)
*Multiparous*	80.40 (279)	73.50 (83)	75.00 (57)
Fetal sex, *% (n)*^b^			
*Female*	54.70 (188)	59.30 (67)	53.90 (41)
*Male*	45.30 (156)	40.70 (46)	46.10 (35)
Infant age at Year 1	N/A	12.00 (1.00)	12.00 (1.00)
Highest education level, *% (n)*^a^			
*Left before GCSE*	5.80 (19)	0 (0)	0 (0)
*GCSE & Vocational*	23.30 (77)	17.30 (19)	13.70 (10)
*A-level*	12.40 (41)	12.70 (14)	12.30 (9)
*University*	32.40 (107)	35.50 (39)	37.00 (27)
*Postgraduate*	26.10 (86)	34.50 (38)	37.00 (27)
Family income, *% (n)*^a^			
*<18,000*	9.00 (30)	1.80 (2)	1.30 (1)
*18 – 25,000*	10.20 (34)	5.40 (6)	3.90 (3)
*25-43,000*	19.20 (64)	19.60 (22)	15.80 (12)
*>43,000*	48.60 (162)	64.30 (72)	69.70 (53)
*Do not wish to say*	12.90 (43)	8.90 (10)	9.20 (7)
Occupation status at Year 1, *% (n)*			
*Working*	N/A	81.30 (91)	73.90 (51)
*Not working*		18.80 (21)	26.10 (18)
WIMD score^a^^,^ᵻ	1216.50 (1242.25)	1487.00 (902.50)	1352.00 (1003.25)
A1 EPDS score^a^	7.00 (6.00)	7.00 (6.75)	6.00 (6.00)
A1 STAI score^a^	34.00 (13.00)	33.00 (13.00)	33.00 (13.00)

WIMD: Welsh Index of Multiple Deprivation; EPDS: Edinburgh Postnatal Depression Scale; STAI: State-Trait Anxiety Inventory

a Data collected at baseline

b Data collected from midewife recorded notes following birth

ᵻ WIMD scores are generated utilising anonymised postcodes (http://wimd.wales.gov.uk). It has a possible range of 1 to 1909, with a low score indicative of an area of higher deprivation and conversely a high score indicative of an area of lower deprivation

### 3.2 Prenatal questionnaire (A1)

Participants completed a questionnaire at their term presurgical appointment incorporating questions on general demographics, lifestyle and mental health. Self-reported depression and anxiety symptoms were assessed via the Edinburgh Postnatal Depression Scale (EPDS) (Cox et al., 1996) and the trait subscale of the Spielberger State-Trait Anxiety Inventory (STAI) (Grant et al., 2008; Meades and Ayers, 2011). Characteristics of the 355 participants who completed the prenatal questionnaire have been reported previously (Janssen et al., 2018).

### 3.3 Year one questionnaire (Y1)

At approximately one year postpartum, 126 participants completed another questionnaire reporting general demographics, EPDS and STAI scores. Participants additionally completed The Postpartum Bonding Questionnaire (PBQ) (Brockington et al., 2006) to assess their perceptions of mother-infant bonding. This 23 item questionnaire consists of three scales. Factor 1 assesses general problems with bonding, e.g. “I feel trapped as a mother”. Factor 2 identifies rejection and pathological anger, e.g. “I feel angry with my baby”. Factor 3 recognises infant-focused anxiety, e.g. “my baby makes me feel anxious”. Factor 4 was not incorporated due to the extreme nature of the questions. The Cardiff Infant Contentiousness Scale (CICS) (Hay et al., 2010) was used to assess the mother's perception of aggressive behaviour (Hay et al., 2014). The Infant Behavioural Questionnaire Revised – Short Form (IBQ-R-SF) (Gartstein, 2003; Rothbart, 1981) was used to assess the mother's perception of infant temperament focusing on three major domains: negative affect, regulatory capacity and surgency (Rothbart, 2011). Four questions from the soothability subscale were inadvertently omitted during questionnaire printing. Of the 126 participants completing the Y1 questionnaire, six were non-Caucasian, three did not ultimately deliver by ELCS, one had a visual impairment, one was not term, one was 18 months and one had no recorded age at questionnaire completion. After these exclusions, data from the remaining 113 questionnaires was analysed. For these participants, 19/113 (16.8%) had scored ≥13 on the EPDS questionnaire and 33/113 (29.2%) had scored ≥40 on the STAI questionnaire that they completed just prior to their term delivery, slightly higher than the overall prevalence in the Grown in Wales Study cohort of 14.3% and 27.3%, respectively (Janssen et al., 2018). Demographic results are presented in Table 1. Missing data was addressed in the EPDS, STAI, PBQ and CICS by utilising participant level mean substitution for participants missing <20% of data. Missing data on the IBQ-R-SF was addressed in line with the IBQ manual.

### 3.4 Infant laboratory assessment

Of the 355 participants recruited to the GiW cohort, 83 attended a laboratory based assessment with their infants. Of these, three participants were non-Caucasian, two did not ultimately deliver by ELCS, one was not born at term and for one assessment the father attended. Demographic data for 76 participants eligible for inclusion in the assessment analysis is provided in Table 1. For these participants, 14/76 (18.4%) had scored ≥13 on the EPDS questionnaire and 20/76 (26.3%) had scored ≥40 on the STAI questionnaire that they completed just prior to their term delivery. Year one questionnaire data was available for 69 of these infants.

#### 3.4.1 Setting

Mothers and infants were assessed by two trained researchers unaware of prenatal EPDS and STAI scores in a designated experimental testing room. Infants were assessed using six components of the Laboratory Temperament Assessment Battery (Lab-TAB) (Goldsmith and Rothbart, 1996): Free Play, Collaboration, Sustained Attention, Maternal Separation, Novel Toy and Joy tasks. For the purpose of this study, three of these tasks were analysed: Sustained Attention, Maternal Separation and Novel Toy.

#### 3.4.2 Novel toy task

Fear was measured using a version of the mechanical toy task from the Lab-TAB manual (Goldsmith and Rothbart, 1996) as previously described (Baker et al., 2012). Infants were sat in a chair and a robot not previously seen by the infant was brought into the room to ensure the novelty effect of the experiment. The mother sat behind the infant and was requested to not interact with the child. The procedure consisted of three trials. In each trial, the robot walked towards the child stopping approximately 20 cm from the child, pausing for ten seconds, walking backwards to the starting point and pausing for a further five seconds. After the third trial the infant was given the opportunity to interact with the robot. If the task was stopped due to the infant (i.e. distress) coding was continued with the score in the last eligible epoch.

#### 3.4.3 Sustained attention task

Sustained attention was measured using a carousel toy. Coding was based on the Lab-TAB repeated visual stimulation task. The procedure had a similar setup to the fear task. The carousel was positioned approximately 40 cm away from the infant, and was set to play for approximately three minutes. The task lasted three minutes or until the infant became distressed and it was not possible for attention to be measured accurately. If the task was stopped due to the infant (i.e. distress) coding was continued with the score in the last eligible epoch.

#### 3.4.4 Maternal separation task

Mothers were instructed to leave the room in a manner they would usually do at home e.g. saying goodbye or just walking out quietly. One experimenter left the room with the mother. One experimenter stayed in the room but did not interact with the infant during a two minute period during which the infant had free range to play with any toy available to them in the room.

#### 3.4.5 Infant neurodevelopment

Age-standardised cognition and language development was measured after the Lab-TAB tasks using the Bayley Scales of Infant Development Third Edition (BSID-III) (Bayley, 2006). Receptive and expressive language were measured during the assessment.

### 3.5 Statistical analysis

All analyses were undertaken using IBM SPSS Statistics Version 25. Normality was assessed via consideration of skewness and kurtosis, normality tests and plots, with all demographic data determined to be non-parametric. For the questionnaire measures, partial correlations controlling for infant age at questionnaire completion were utilised to assess the relationship between prenatal depression and anxiety symptoms and maternally self-reported mood, bonding and infant outcomes at year one. To determine the potential influence of infant gender, this analysis was repeated separately for male and female infants. For relationships significant at p<.05, multiple linear regression was employed to determine if this relationship remained when controlling for both infant age and for parity, a factor previously suggested to influence early mother-infant interactions (Fish and Stifler, 1993). The same analyses were then undertaken for the assessment measures, but controlling for infant age at assessment, to determine the relationship between prenatal anxiety and depression and objectively assessed infant development and temperament. The assessment analysis was repeated as outlined above utilising year one postnatal depression and anxiety symptom scores.

## 4 Results

### 4.1 Questionnaire analysis for all 113 mother-infant dyads

Partial correlations were utilised to assess the relationship between maternally reported depression and anxiety symptoms reported just prior to a term delivery and maternally reported mood symptoms one year later, infant bonding and infant outcomes, controlling for infant age at questionnaire completion (Table 2). There was a significant relationship between term EPDS and STAI scores and year one EPDS and STAI scores, components of the PBQ, the CICS and components of negative affect and regulatory capacity subscales on the IBQ-R-SF.

**Table 2:** Partial correlations between term EPDS and STAI scores and maternally reported mood, maternal bonding and infant outcomes at year one, controlling for infant age at questionnaire completion.

Questionnaire measure	n	Median	Term Depressive symptoms		Term Anxiety symptoms	
			*r^s^*	*p*	*r^s^*	*p*
Y1 EPDS total	112	6.00	**.63**	**<.001**	**.57**	**< .001**
Y1 STAI total	112	33.00	**.65**	**<.001**	**.75**	**< .001**
PBQ						
*PBQ factor 1*	113	4.00	**.32**	**.001**	**.24**	**.012**
*PBQ factor 2*	113	1.00	.17	.083	.17	.071
*PBQ factor 3*	113	2.00	**.19**	**.048**	**.34**	**< .001**
CICS	112	7.00	**.27**	**.004**	**.24**	**.013**
IBQ-R-SF						
Negative Affect	111	3.60	**.30**	**.002**	**.26**	**.006**
*Sadness*	111	3.67	**.30**	**.002**	**.30**	**.002**
*Distress*	111	4.14	**.26**	**.007**	**.26**	**.007**
*Fear*	111	3.50	.09	.339	.07	.495
*Falling Reactivity*	111	5.67	**-.21**	**.030**	-.14	.156
Surgency	111	5.43	.10	.308	-.03	.741
*Approach*	111	6.17	-.11	.237	-.10	.284
*Vocal reactivity*	111	5.71	.18	.068	.05	.610
*High intensity pleasure*	111	6.71	-.02	.865	-.18	.068
*Smiling*	111	5.43	.12	.215	-.09	.349
*Activity*	111	4.29	.16	.101	.10	.326
*Sensitivity*	111	5.17	.00	.976	.00	.967
Regulatory Capacity	111	4.95	-.04	.655	-.08	.383
*Low intensity pleasure*	111	4.14	.03	.776	-.01	.913
*Cuddliness*	111	5.00	-.07	.471	-.11	.277
*Orienting*	111	4.17	.16	.090	.16	.094
*Soothability*	111	5.67	**-.26**	**.007**	**-.28**	**.003**

Y1: Year 1; EPDS: Edinburgh Postnatal Depression Scale; STAI: State-Trait Anxiety Inventory; PBQ: Postpartum Bonding Questionnaire; CICS: Cardiff Infant Contentiousness Scale; IBQ-R-SF: Infant Behavioural Questionnaire – Revised – Short Form.

### 4.2 Questionnaire analysis for mother-infant dyads by infant sex

To ascertain whether infant sex contributed to the mother's perceptions, questionnaire data was analysed for boys (n = 46) and girls (n = 67) separately. Table 3 shows the partial correlations between prenatal EPDS and STAI scores and maternally rated mood and infant temperament, again controlling for infant age at questionnaire completion, separate for male and female infants. This analysis revealed that the perceptions of mothers of boys were different to the perceptions of mothers of girls in relation to their self-reported symptoms of prenatal depression and anxiety. Significant relationships for components of the PBQ, CICS and the IBQ-R-SF were identified with female infants whereas minimal association were found with the male infant data.

**Table 3:** Partial correlations between term EPDS and STAI scores and maternally reported mood, maternal bonding and infant temperament at year one for male and female infants controlling for infant age at questionnaire completion.

	Term Depressive symptoms				Term Anxiety symptoms			
Questionnaire measure	Females		Males		Females		Males	
	*r^s^*	*p*	*r^s^*	*p*	*r^s^*	*p*	*r^s^*	*p*
Y1 EPDS total	**.63**	**< .001**	**.61**	**< .001**	**.52**	**< .001**	**.64**	**< .001**
Y1 STAI total	**.68**	**< .001**	**.62**	**< .001**	**.76**	**<.001**	**.73**	**< .001**
PBQ								
*PBQ factor 1*	**.49**	**< .001**	.02	.916	**.26**	**.035**	.16	.310
*PBQ factor 2*	.23	.074	.03	.847	.11	.397	.24	.115
*PBQ factor 3*	**.26**	**.042**	.09	.542	**.40**	**.001**	.25	.104
CICS	**.37**	**.003**	.10	.525	.24	.055	.24	.117
IBQ-R-SF								
Negative Affect	**.30**	**.016**	.25	.102	**.25**	**.047**	.25	.097
*Sadness*	**.38**	**.002**	.14	.359	**.32**	**.010**	.26	.093
*Distress*	**.32**	**.011**	.08	.592	**.30**	**.016**	.14	.356
*Fear*	-.07	.594	.26	.082	-.07	.595	.19	.228
*Falling Reactivity*	-.25	.052	-.16	.285	-.17	.178	-.10	.528
Surgency	.20	.118	-.01	.973	.09	.479	-.18	.255
*Approach*	.06	.665	**-.30**	**.047**	-.01	.914	-.21	.170
*Vocal reactivity*	.13	.312	**.30**	**.044**	.05	.684	.10	.531
*High intensity pleasure*	.09	.502	-.07	.656	-.05	.677	-.27	.077
*Smiling*	.10	.442	.16	.301	-.12	.337	-.05	.752
*Activity*	.22	.085	.05	.745	.18	.149	-.04	.805
*Sensitivity*	.16	.195	-.17	.273	.19	.137	-.19	.230
Regulatory Capacity	-.04	.783	-.04	.789	-.08	.527	-.10	.524
*Low intensity pleasure*	.07	.597	.00	.990	.026	.839	-.05	.759
*Cuddliness*	-.07	.581	-.08	.586	-.13	.318	-.09	.557
*Orienting*	.21	.098	.11	.494	.15	.239	.16	.302
*Soothability*	**-.31**	**.013**	-.16	.301	**-.26**	**.035**	-.30	.054

Y1: Year 1; EPDS: Edinburgh Postnatal Depression Scale; STAI: State-Trait Anxiety Inventory; PBQ: Postpartum Bonding Questionnaire; CICS: Cardiff Infant Contentiousness Scale; IBQ-R-SF: Infant Behavioural Questionnaire – Revised – Short Form.

### 4.3 Multiple linear regression of questionnaires controlling for infant age and parity

For variables significant at *p* < .05 in Table 3, multiple linear regression was used to assess the predictive nature of the relationship and establish if the relationship remained when controlling for both infant age at questionnaire completion and parity, which may play a moderating role. With the exception of Vocal reactivity on the IBQ-R-SF, all identified relationships with prenatal maternal EPDS scores remained significant. Prenatal EPDS scores were significantly associated with Year 1 EPDS (*p* < .001, B = .70, 95% CI .43, .98) and Year 1 STAI scores (*p <* .001, B = 1.53, 95% CI .92, 2.15) for mothers with male infants, and with Year 1 EPDS (*p* < .001, B = .58, 95% CI .40, .76) and Year 1 STAI (*p* < .001, B = 1.41, 95% CI 1.02, 1.80) scores for mothers with female infants.

For mothers with female infants, prenatal EPDS scores were significantly associated with PBQ factor 1 (*p <* .001, B = .39, 95% CI .22, .56) and PBQ factor 3 (*p =* .041, B = .10, 95% CI .00, .19). For female infants, prenatal depression symptoms were significantly associated with the CICS (*p =* .003, B = .19, 95% CI .07, .31) and on the IBQ-R-SF; Negativity (*p =* .017*,* B = .05, 95% CI .01, .09), Sadness (*p* = .002, B = .08, 95% CI .03, .13), Distress (*p =* .012, B = .08, 95% CI .02, .13) and Soothability (*p =* .012, B = -.06, 95% CI -.10, -.01). For male infants, prenatal EPDS scores remained significant only with Approach on the IBQ (*p =* .043, B = -.04, 95% CI -.09, .00).

When controlling for both infant age at questionnaire completion and parity, with the exception of Negativity, all significant relationships with prenatal STAI scores remained. That is, prenatal STAI scores were significantly associated with Year 1 EPDS (*p <* .001, B = .39, 95% CI .24, .54) and Year 1 STAI scores (*p <* .001, B = .97, 95% CI .68, 1.25) for mothers with male infants, and with Year 1 EPDS (*p <* .001, B = .27, 95% CI .15, .38) and Year 1 STAI (*p* <.001, B = .90, 95% CI .70, 1.09) scores for mothers with female infants. For mothers with female infants, on the IBQ-R-SF prenatal anxiety symptoms were significantly associated with Sadness (*p =* .012, B = .04, 95% CI .01, .07), Distress (*p =* .017, B = .04, 95% CI .01, .07) and Soothability (*p =* .039, B = -.03, 95% CI -.05, .00).

### 4.4 Laboratory assessment for all mother-infant dyads

Partial correlations were utilised to determine the relationship between prenatal EPDS and STAI scores and assessments of infant development and temperament through the BSID-III and Lab-TAB tasks, controlling for infant age at assessment (Table 4). Maternal prenatal EPDS scores were significantly correlated with cognitive scores on the BSID-III and one element of the novel toy task. Maternal prenatal STAI scores were significantly correlated with receptive language. Both prenatal EPDS and STAI scores were significantly associated with one element of the maternal separation task.

**Table 4:** Partial correlations between term depression and anxiety symptoms and assessed infant outcomes at one year, controlling for infant age at assessment.

Assessment measure	n	Median	Term depressive symptoms		Term anxiety symptoms	
			r^s^	p	r^s^	p
BSID-III						
*Cognitive*	76	17.00	**-.31**	**.021**	-.19	.160
*Receptive language*	*60*	11.00	-.26	.052	**-.31**	**.018**
*Expressive language*	*58*	13.00	-.16	.232	-.22	.102
Lab-TAB						
Novel toy (fear)						
*Facial fear*	*67*	.80	.10	.439	-.07	.560
*Distress*	*67*	.30	.13	.310	-.07	.602
*Bodily fear*	*67*	.20	.08	.534	-.08	.549
*Intensity of escape*	*67*	.20	**.26**	**.035**	.10	.442
*Startle response*	*67*	.00	-.09	.489	-.14	.268
*Parent behaviour*	*66*	.00	-.08	.536	-.19	.127
Sustained attention						
*Facial interest*	*69*	.80	-.20	.112	-.17	.189
*Duration of looking*	*69*	22.20	-.08	.529	-.08	.516
*Gestures*	*69*	.10	.00	.965	.07	.577
*Parent behaviour*	*68*	.30	-.23	.072	-.13	.312
*Infant positive affect*	*67*	.30	.06	.634	-.04	.738
*Infant negative affect*	*69*	.30	-.04	.747	.01	.947
*Latency to look away*	*68*	4.00	-.05	.703	.04	.777
Maternal separation						
*Facial fear*	*50*	.75	.14	.349	.19	.189
*Distress*	*70*	.85	**-.29**	**.042**	**-.30**	**.036**
*Latency to fear response*	*70*	1.00	-.13	.365	.02	.906
*Bodily fear*	*62*	.30	-.12	.431	-.09	.549
*Escape*	*70*	.45	-.12	.395	-.08	.610

BSID-III: Bayley Scales of Infant Development Third Edition; Lab-TAB: Laboratory Temperament Assessment Battery

### 4.5 Laboratory assessment analysis analysed by infant sex

To assess the potential influence of infant sex on this relationship, data was analysed separately for males and females, again using partial correlations controlling for infant age at assessment (Table 5). The objective assessments identified significant relationships between maternal prenatal EPDS and STAI scores and infant temperament and development primarily for male and not female infants.

**Table 5:** Partial correlations between term EPDS and STAI scores and independently assessed infant outcomes at one year by gender, controlling for infant age at assessment.

Assessment measure	Term Depressive symptoms				Term Anxiety symptoms			
	Females		Males		Females		Males	
	*r^s^*	*p*	*r^s^*	*p*	*r^s^*	*p*	*r^s^*	*p*
BSID-III								
*Cognitive*	-.34	.079	-.26	.184	-.24	.213	-.12	.543
*Receptive language*	-.17	.398	-.34	.075	-.23	.244	**-.41**	**.032**
*Expressive language*	.07	.735	**-.44**	**.018**	-.02	.925	**-.49**	**.008**
Lab-TAB								
Novel toy (fear)								
*Facial fear*	.30	.073	-.18	.367	.10	.574	-.32	.102
*Distress*	.30	.072	-.15	.458	.06	.751	-.26	.182
*Bodily fear*	.27	.107	-.11	.580	.11	.534	-.29	.149
*Intensity of escape*	**.39**	**.018**	.02	.914	.20	.252	-.09	.643
*Startle response*	.04	.821	-.20	.304	-.07	.701	-.22	.262
*Parent behaviour*	.08	.631	-.25	.196	-.02	.888	**-.39**	**.044**
Sustained attention								
*Facial interest*	-.03	.889	**-.44**	**.015**	.03	.855	**-.39**	**.038**
*Duration of looking*	.03	.878	-.18	.330	.05	.771	-.19	.316
*Gestures*	.00	.998	-.09	.632	.05	.791	.04	.854
*Parent behaviour*	-.25	.170	-.24	.206	.03	.882	-.25	.188
*Infant positive affect*	.10	.576	.02	.917	-.05	.776	-.03	.866
*Infant negative affect*	-.14	.444	.01	.946	.01	.948	-.03	.872
*Latency to look away*	.11	.557	-.18	.342	.20	.257	-.08	.665
Maternal separation								
*Facial fear*	-.14	.480	**.48**	**.039**	-.07	.719	**.67**	**.002**
*Distress*	-.24	.214	-.34	.155	-.22	.263	-.44	.070
*Latency to fear response*	.00	.983	-.34	.152	.05	.803	-.37	.126
*Bodily fear*	-.15	.448	-.17	.482	-.10	.607	-.20	.429
*Escape*	-.08	.688	-.21	.379	-.06	.773	-.15	.550

BSID-III: Bayley Scales of Infant Development Third Edition; Lab-TAB: Laboratory Temperament Assessment Battery

### 4.6 Multiple linear regression of assessment data controlling for infant age and parity

Multiple linear regression was utilised to establish if the relationships significant at *p* < .05 in Table 5, remain significant when controlling for age at assessment and parity, and the predictive nature of this association. After adjustment, with the exception of facial interest in the sustained attention task and prenatal anxiety symptoms, all identified relationships remained significant. For male infants only, term EPDS scores were significantly associated with expressive language on the BSID-III (*p =*.009, B = -.19, 95% CI -.33, -.05), facial interest on the sustained attention task (*p =* .023, B = -.02, 95% CI -.03, .00) and facial fear on the maternal separation task (*p =* .039, B = .09, 95% CI .00, .17). For male infants only, term STAI scores remained significantly associated with receptive (*p =* .024, B = -.08, 95% CI -.14, -.01) and expressive (*p =* .005, B = -.11, 95% CI -.17, -.04) language on the BSID-III, parent behaviour on the novel toy task (*p =* .044, B = -.02, 95% CI -.05, .00) and facial fear on the maternal separation task (*p =* .003, B = .08, 95% CI .00, .03). For female infants only, there was a significant association between term EPDS scores only with intensity of escape on the novel toy task (*p =* .024, B = .05, 95% CI .01, .09).

### 4.7 Multiple linear regression of Year 1 EPDS and STAI scores and assessment data controlling for infant age and parity

The analysis was repeated to establish the relationships between maternal mood scores at Year 1 and the infant assessment data (Table S1). After adjusting for age at assessment and parity, for male infants Year 1 EPDS scores were associated with facial interest on the sustained attention task (*p* = .001, B = -.02, 95% CI = -.03, -.01) and no other measure. Year 1 STAI scores were associated with receptive language (*p* = .040, B = -.06, 95% CI = -.12, .00), expressive language (*p* = .042, B = -.07, 95% CI = -.13, .00) and facial interest in the sustained attention task (*p* = .007, B = -.01, 95% CI = -.02, .00). There was no relationship between either EPDS or STAI scores and any female infant characteristic.

## 5 Discussion

The major finding of this paper is that prenatal symptoms of depression and anxiety were associated with maternal perceptions of negative temperament and behaviour in daughters but not sons one year after birth. Conversely, in objective laboratory based assessments prenatal mental health symptoms were associated with difficulties in language development and temperament in male infants with minimal associations between the mothers’ mood and female infant characteristics. These data support the prevalent theory that males exposed in utero to maternal mood symptoms are biologically impacted very early in life and further suggest that difficulties may not be recognised by affected mothers. In contrast, mood affected mothers recognised difficulties in female infants which were less apparent to researchers.

Numerous studies have reported the link between maternal mood symptoms in pregnancy and maternal perceptions of impaired bonding with infants (Dubber et al., 2015). Consistent with these previous studies, in our study self-reported symptoms of prenatal anxiety and depression obtained just prior to a term delivery were associated with maternally rated measures of infant bonding. Mothers reporting higher symptoms of prenatal depression or anxiety reported significantly more difficulties in PBQ subscales reflective of impaired bonding (PBQ1) and feelings of infant focused anxiety (PBQ3). However, when the data was reanalysed separately for male and female infants, and after controlling for infant age and parity, these associations remained significant only for the female infants. Poor maternal bonding of anxious mothers with their daughters has been reported previously (Muller et al., 2016). Depression has been shown to exhibit strong matrilineal transmission patterns and the mother-daughter brain circuits associated with emotion regulation are more correlated relative to mother-son (Yamagata et al., 2016). Poor mother-infant bonding would be consistent with the higher prevalence of depression symptoms in teenage daughters of depressed and anxious mothers (Quarini et al., 2016; Van den Bergh et al., 2008). Consistent with previous studies (Hay et al., 2011), mothers reporting higher symptoms of depression also reported significantly higher aggression in their infants, but only when the infant was female. Insecure attachment has been suggested as one factor influencing the development of aggression in infants (Jones et al., 2015) which would be consistent with the maternal reporting of suboptimal bonding specifically with female infants. Negative affect, which describes a wide range of negative emotions including both fearfulness, irritability and frustration, is the temperament most commonly associated with prenatal depression and anxiety (Austin et al., 2005; Glynn et al., 2018; McGrath et al., 2008; Spry et al., 2019). We similarly identified a significant positive association between prenatal depression and anxiety symptoms and maternally reported infant temperament for the subcomponents of sadness and distress but only when the infant was female. Overall, the mood affected mothers had more negative perceptions of their daughters in a number of domains.

In contrast to the data for girls, mood affected mothers of boys reported no concerns around bonding and minimal concerns with their son's behaviour. Only the association between prenatal depression symptoms and the surgency subdomain “approach” was significant. This could simply mean that our numbers for boys were too low to detect associations. Alternatively, these findings may relate to sex difference in maternal perceptions impacted by maternal mood. For example, perceptions may be influenced by maternal expectations based on the infant's sex. Being an “easy” or “difficult” child may depend on whether they are male or female. Alternatively, mothers may expect to feel closer to their female infants or may expect their daughters to behave more considerately than their sons. When these gendered expectations are not met, the depressed or anxious mother may perceive difficulties which are not present. A third possibility is that depressed or anxious mothers are sensitive to their daughters’ difficulties but not to their sons.

Assessments by researchers play a critical role in providing unbiased data not influenced by gendered expectations (Whiffen and Gotlib, 1989). In our objective assessment of infant development and temperament conducted by trained researchers, we were able to identify associations between mood symptoms and specific infant characteristics. These associations were most evident with respect to the language development of boys, and their facial responses in certain tasks. Prenatal anxiety symptoms and anxiety symptoms reported at the time of the assessment were both significantly associated with lower scores for receptive and expressive language. Prenatal depression symptoms, but not symptoms reported at the time of the assessment, were associated with lower scores for expressive language. Boys had difficulties in both understanding and using spoken language which may underlie their mothers’ reports of concerns around approach behaviour as the ability to communicate is closely linked to social skills. Prenatal depression and anxiety symptoms negatively correlated with facial interest in the sustained attention task and positively associated with higher scores for facial fear in the maternal separation task. Facial interest is a measure of ability to sustain attention and boys raised by anxious mothers display higher levels of inattention (O'Connor et al., 2002). Facial fear could suggest insecure attachment which has been reported for boys exposed to maternal depression (Murray et al., 2011). Mothers of boys reporting prenatal anxiety symptoms were found to be more likely to interfere in the novel toy task. However, year one anxiety scores were not associated with parent behaviour or facial fear. Together, these data indicate that boys of mothers with mood symptoms are limited in their ability to verbally communicate. This may underlie a higher reliance on non-verbal communication via facial expressions. Given the close correlation between maternal mood scores at the two times points, however, we cannot conclude that the prenatal mood symptoms are having a greater impact on the male infant than the continued exposure to postnatal symptoms.

In contrast to the boys, analysis of the Lab-TAB findings for female infants identified an association only between prenatal depression symptoms and intensity of escape during the novel toy task, and there were no associations between maternal mood symtoms at Year 1 and the female infant characteristics assessed. Intensity of escape is a measure of fear response and has been linked to the development of anxiety (Buss, 2011). A greater risk of anxiety for teenage girls exposed to maternal depression has previously been reported (Murray et al., 2011). The absence of other correlations for female infants were not simply due to the small sample size as the numbers were similar for male and female infants, and we obtained significant findings for males with these numbers. Moreover, analysis of the combined data revealed fewer overall associations between maternal mood symptoms and infant characteristics despite the larger number of participants indicating that combining data from males and females was masking significant associations with mood. These findings do not exclude an effect of maternal mood on female infants. These may manifest at a later age or with different tests. Indeed, several studies have reported adverse outcomes for girls as well as boys later in childhood, and in adolescence and adulthood (Glover and Hill, 2012). It is also possible that the mood-affected mother's more negative perceptions of their female infants may underlie the difficulties these girls experience later in life rather than exposure during the pregnancy. Nonetheless, these findings do lend further support to the hypothesis that maternal mood symptoms have a greater impact on male infants early on, with boys developing more difficulties than girls particularly in relation to language. Importantly, these difficulties were not being recognised by their mood affected mothers.

The mechanisms linking exposure to sexually dimorphic outcomes are unknown. Differences in the behavioural outcomes between boys and girls born to depressed and anxious mothers may be due to differences in sex hormone exposures (McEwen and Milner, 2017) or differences in the development of the hypothalamus-pituitary-adrenocortical axis (Carpenter et al., 2017). There are also intrinsic epigenetic differences between males and females (Li et al., 2018). Sex differences in DNA methylation at X chromosome and autosomal sites have been reported, some with differential trajectories of DNA methylation in the developing male and female brain (Spiers et al., 2015). Sex differences in DNA methylation also exist in the placenta (Martin et al., 2017; Sood et al., 2006). We have previously reported an association between both clinically diagnosed and self-reported symptoms of prenatal depression and reduced expression of the epigenetically-regulated gene *PEG3* in male placenta and not female placenta (Janssen et al., 2016) highlighting the potential for epigenetic differences between males and females. In this respect, it is interesting to note that higher DNA methylation at *PEG*3, predicted to lower gene expression, has been positively related to negative affectivity in another study (Fuemmeler et al., 2016). A further possibility worth exploring is the societal expectations placed on children. Stereotypical beliefs that boys and girls differ in their emotional and cognitive abilities are deeply embedded in our society (Raymond, 2013). Stereotyping can affect parental behaviour from the earliest interactions with the infant, with baby boys and girls effectively being colour coded (Stern and Karraker, 1989) and treated differently based on gendered clothing (Leone and Robertson, 1989). Maternal perceptions of difficult temperament may depend, in part, on expectations based on the sex of the infant (Condry and Condry, 1976) contributing to sex-differences in outcomes.

### 5.1 Strengths

The focused nature of our cohort is both a strength and a limitation of the study. Participants were all recruited by the same two research midwives following a specific protocol prior to a term delivery, completing the questionnaire at the same time of day. All babies were delivered at term (after 37 weeks) by a booked ELCS. All the mothers whose data were analysed were Caucasian with a majority of Welsh upbringing (Janssen et al., 2018). The strict criteria applied in our recruitment and analysis was intended to generate a relatively homogeneous cohort for epigenetic studies, which will be reported elsewhere. This relative homogeneity may explain why we were able to reveal striking correlations both with respect to questionnaire data and our laboratory infant findings despite the relatively small number of participants. Importantly, our findings are not spurious because splitting by sex, effectively halving the number of participants in each analysis, revealed more significant correlations than were found when combining data from both sexes.

### 5.2 Limitations

The clear limitation of our study is relevance to other modes of delivery, populations and ethnicities. 91% of our original GiW study participants are Caucasian (Janssen et al., 2018). We analysed data from only these participants due to the low numbers of non-Caucasian participants. All our infants were delivered by ELCS. ELCS substantially differs from other modes of delivery as mothers do not undergo the physiological process of labour which may have longer term consequences for their mood (Janssen et al., 2018). We have not identified another study which has analysed infant language development and temperament in the context of maternal mood symptoms specifically for infants delivered by ELCS for comparison. Although we can find no reason why mode of delivery would drive sex differences in outcome, we cannot exclude the possibility that sex differences in maternal perceptions of infants or their characteristics are impacted by mode of delivery.

A further limitation is that we have measurements of maternal mood by self-report. We cannot be sure that our maternal measures accurately reflect degree of exposure but prenatal mood scores were highly correlated with year one mood scores both for the EPDS and the STAI questionnaires and maternal mood symptoms in pregnancy highly correlated with mental health history in our cohort (Janssen et al., 2018). Higher mood scores prenatally and at year one suggests continued mood symptoms through the perinatal period. Both prenatal and postnatal mood symptoms are associated with adverse outcomes (Lahti et al., 2017; Raikkonen et al., 2015; Talge et al., 2007; Van den Bergh et al., 2005). Although the prenatal mood scores were significantly associated with more infant characteristics than the year one scores, we cannot attribute our findings to a specific time of exposure or solely to depression or anxiety due to their pronounced correlations. Some of the participants were prescribed antidepressants and it will be important going forward to understand how these drugs impact both the gendered perceptions and the laboratory assessments of boys and girls in larger cohorts with sufficient numbers to tease out differences between untreated and treated depression, and the relevance of the timing of exposures. We acknowledge that our study size was small and it will be important to validate our findings in a larger cohort, and to disentangle these relationships.

Finally, Lab-TAB offers advantages over subjective maternal reporting as clearly evidenced by this study. However, measurements in the Lab-TAB were not directly comparable to the maternally reported characteristics. We sampled limited components of the infant's behavioural repertoire. Tests were also performed in a novel environment and home assessment may uncover characteristics not observed in a research setting.

## 6 Conclusions

Our data is consistent with the growing body of evidence that males are less resilient to early life stressors than females (Sutherland and Brunwasser, 2018). The novel finding of our study was that mothers reporting higher prenatal depression and anxiety symptoms reported negative characteristics for their infant only when the infant was female, and not when the infant was male. In contrast, our objective assessment uncovered an impact of maternal mood symptoms primarily on male infants with delays in language development and differences in facial responses, whereas female infants appeared relatively unaffected. This study supports the vulnerability of males early in life to stressors and raises the intriguing possibility that females are resilient early in life but may develop issues later in life as a consequence of poor bonding with their mood affected mother. Importantly, the biased perceptions of mood affected mothers of their sons’ difficulties may delay the identification and targeted support of these children at a most critical time.

## Contributors

Conceptualisation RMJ, SvG, ABJ; data curation ABJ, JSK, KAS, LAS, SMG, KD; formal analysis KAS and SMG; supervision RMJ; Wrt writing original draft RMJ, KAS, SMG and SvG. Funding acquisition RMJ, SvG. All authors have approved the final article.

## CRediT authorship contribution statement

**K. Savory:** Data curation, Formal analysis, Writing - original draft. **S.M. Garay:** Data curation, Formal analysis, Writing - original draft. **L.A. Sumption:** Data curation. **J.S. Kelleher:** Data curation. **K. Daughters:** Data curation. **A.B. Janssen:** Conceptualization, Data curation. **S. Van Goozen:** Conceptualization, Funding acquisition, Writing - original draft. **R.M. John:** Conceptualization, Funding acquisition, Supervision, Writing - original draft.

## Declaration of Competing Interest

None.

## Appendix Supplementary materials

Image, application 1

Image, application 2
