# Supplementary material for: Prenatal symptoms of anxiety and depression associated with sex differences in both maternal perceptions of one year old infant temperament and researcher observed infant characteristics
Source: J Affect Disord. 2020 Mar 1;264:383–92. doi: 10.1016/j.jad.2019.11.057 (PMC7005670; doi:10.1016/j.jad.2019.11.057)
Supplement: Supplementary file 1 [file mmc1.docx]

**Table S1**. Partial correlations between Year 1 EPDS and STAI scores and independently assessed infant outcomes at one year by gender, controlling for infant age at assessment.

| **Assessment measure** | | **Term Depressive symptoms** | | | | **Term Anxiety symptoms** | | | |
| --- | --- | --- | --- | --- | --- | --- | --- | --- | --- |
|  | | **Females** | | **Males** | | **Females** | | **Males** | |
|  | | *r^s^* | *p* | *r^s^* | *p* | *r^s^* | *p* | *r^s^* | *p* |
| BSID-III | |  |  |  |  |  |  |  |  |
|  | *Cognitive* | .13 | .512 | -.32 | .158 | -.02 | .932 | -.29 | .209 |
|  | *Receptive language* | -.10 | .633 | -.26 | .260 | -.14 | .465 | **-.45** | **.041** |
|  | *Expressive language* | .17 | .411 | -.41 | .063 | .06 | .747 | **-.45** | **.041** |
|  |  |  |  |  |  |  |  |  |  |
| Lab-TAB | |  |  |  |  |  |  |  |  |
| Novel toy (fear) | |  |  |  |  |  |  |  |  |
|  | *Facial fear* | .26 | .127 | -.24 | .253 | .29 | .091 | -.38 | .076 |
|  | *Distress* | .15 | .380 | -.03 | .882 | .18 | .305 | -.24 | .263 |
|  | *Bodily fear* | .27 | .118 | -.17 | .429 | .28 | .097 | -.30 | .172 |
|  | *Intensity of escape* | .15 | .379 | .06 | .800 | .23 | .177 | -.12 | .576 |
|  | *Startle response* | .17 | .345 | -.09 | .677 | .04 | .805 | -.18 | .421 |
|  | *Parent behaviour* | .08 | .630 | -.11 | .602 | .20 | .255 | -.219 | .314 |
|  |  |  |  |  |  |  |  |  |  |
| Sustained attention | |  |  |  |  |  |  |  |  |
|  | *Facial interest* | .02 | .907 | **-.72** | **<.001** | -.03 | .886 | **-.60** | **.003** |
|  | *Duration of looking* | .19 | .293 | -.35 | .103 | .10 | .599 | -.28 | .211 |
|  | *Gestures* | -.04 | .822 | -.24 | .268 | -.10 | .597 | -.09 | .686 |
|  | *Parent behaviour* | -.15 | .421 | -.34 | .114 | -.02 | .909 | -.31 | .155 |
|  | *Infant positive affect* | .00 | .990 | -.02 | .935 | -.10 | .594 | -.24 | .291 |
|  | *Infant negative affect* | .02 | .927 | -.01 | .968 | .02 | .930 | .00 | .987 |
|  | *Latency to look away* | -.13 | .479 | -.16 | .454 | -.11 | .541 | -.15 | .513 |
|  |  |  |  |  |  |  |  |  |  |
| Maternal separation | |  |  |  |  |  |  |  |  |
|  | *Facial fear* | .03 | .873 | .12 | .666 | -.20 | .302 | .32 | .239 |
|  | *Distress* | -.26 | .179 | -.13 | .626 | -.29 | .126 | -.30 | .281 |
|  | *Latency to fear response* | .01 | .943 | -.28 | .300 | .18 | .349 | -.28 | .306 |
|  | *Bodily fear* | -.03 | .894 | -.12 | .659 | -.12 | .547 | .00 | .989 |
|  | *Escape* | -.08 | .684 | .08 | .767 | -.07 | .734 | -.21 | .445 |

BSID-III: Bayley Scales of Infant Development Third Edition; Lab-TAB: Laboratory Temperament Assessment Battery

**The above significant correlations remained significant when controlling for parity in addition to age at assessment:**

**Males:**

Y1 STAI + Receptive language: *p* = .040, B = -.06, 95% CI = -.12, .00

Y1 STAI + Expressive language: *p* = .042, B = -.07, 95% CI = -.13, .00

Y1 STAI + Facial interest on Sustained attention task: *p* = .007, B = -.01, 95% CI = -.02, .00

Y1 EPDS + Facial interest on Sustained attention task: *p* = .001, B = -.02, 95% CI = -.03, -.01
